# Interpreting physical sensations to guide health-related behavior: An introductory review on psychosomatic competence

**DOI:** 10.1007/s00508-021-01988-8

**Published:** 2021-12-10

**Authors:** Christian Fazekas, Dennis Linder, Franziska Matzer, Josef Jenewein, Barbara Hanfstingl

**Affiliations:** 1 Department of Medical Psychology and Psychotherapy, Medical University of Graz, Auenbruggerplatz 3, 8036 Graz, Austria; 2 Ben Gurion University of the Negev, Beer-Sheva, Israel; 3 University of Klagenfurt, Klagenfurt am Wörthersee, Austria

**Keywords:** Autoregulation, Cognitive ability, Health behavior, Interoception, Self-regulation

## Abstract

From a biopsychosocial perspective, maintaining health requires sufficient autoregulatory and self-regulatory capacity to both regulate somatic physiology and manage human-environment interactions. Increasing evidence from neuroscientific and psychological research suggests a functional link between so called interoceptive awareness and self-regulatory behavior. Self-regulation can, again, influence autoregulatory patterns as it is known from biofeedback training or meditation practices. In this review, we propose the psychosomatic competence model that provides a novel framework for the interrelation between interoceptive and self-regulatiory skills and health behavior. The term psychosomatic competence refers to a set of mind- and body-related abilities which foster an adequate interpretation of interoceptive signals to drive health-related behavior and physical well-being. Current related empirical findings and future directions of research on interoception and self-regulation are discussed.

## Introduction

Health-related behavior significantly influences health, disease and all-cause mortality [1]. There is considerable evidence that interventions to achieve an at least temporary behavioral change are often effective; conversely, maintenance of an improved health-related behavior may represent a challenging task and frequently leads to limited, if any success [2]. The reasons for these difficulties, although broadly discussed, remain insufficiently understood [3]. In behavioral sciences, theoretical models for maintenance of behavior change are manifold. In a systematic review on this topic, exactly 100 different behavior theories were included and the following core concepts were reported: motives, self-regulation, resources (psychological and physical), habits and environmental and social influences [4]. Within a neuroscientific perspective, increasing evidence suggests that mental health and physical health are linked by neural systems that jointly regulate somatic physiology and high-level cognition [5, 6]. These neural systems are assumed to help “construct” models of the self-in-context by compressing information across time and sensory modalities into conceptions of the underlying causes of experience and thus contribute to “meaning-making” and decision-making. Self-in-context models guide learning from experience and the formation of narratives about the self and one’s world. They integrate perceptual information across exteroceptive and interoceptive senses with conceptual information from memory and allow the subject to understand incoming sensory signals as clues to one’s current state. Self-in-context models are assumed to guide behavior and physiological regulation on the basis of predictive codes [5, 7].

In analogy to self-in-context models, the concept of psychosomatic competence (PSC) assumes a cognitive human ability that allows elaborating essential clues provided by interoceptive signals and subsequent development of body-related self-regulatory behavior in support of somatic physiological processes and maintenance of health. This short review is an introduction to PSC. The concept of PSC allows a novel theoretical approach to the link between interoception, associated cognitive factors and body-related self-regulation. It was developed in a clinical context aiming to provide a comprehensive framework to better understand health-related behavior and coping with burdensome physical sensations. It has only been recently introduced in combination with a new assessment tool, termed the psychosomatic competence inventory (PSCI) [8]. In this review, the underlying theoretical assumptions and components of the concept of PSC as well as the way they are related to other theories and models are outlined. This should help to highlight conceptual overlaps or differences regarding the same or similar terminology in other contexts and is intended to reduce the risk of conceptual confusion, due to jingle-jangle fallacies [9]. We also report on first empirical evidence for the concept of psychosomatic competence and finally point to current research gaps on this matter.

## Psychosomatic competence

It has to be noted that the term psychosomatic competence is used in more than one meaning. In its probably more common understanding, psychosomatic competence indicates the knowledge and skills of a medical professional to explore, understand and deal with biopsychosocial aspects of disease when interacting with patients [10]. In this review, however, the term psychosomatic competence refers to the complete set of body-related abilities and skills, which define psychosomatic intelligence but which are unrelated to traditional intelligence test scores [8, 11, 12]. Psychosomatic competence assumes a basic human ability to react consciously and adequately to perceived body signals by means of body-related and health-related behavior. Examples range from meeting basic physiological needs like maintaining an adequate body temperature, satisfying thirst or hunger, to more complex behavioral patterns, like optimizing one’s feeling of physical well-being, e.g., by reacting to a desire for exercise or by consciously changing an uncomfortable or unhealthy posture during working hours or by adjusting the sleep-wake cycle. Further examples for this competence range from evidence-based biofeedback training for stress or pain reduction purposes to establishing healthy behavioral patterns by conscious attention and reaction to bodily signals, e.g., regarding eating habits.

From a biopsychosocial perspective on health and disease [13], maintenance of health requires sufficient autoregulatory (i.e., unconscious) and self-regulatory (i.e., conscious) capacity to adequately manage the interaction between individual and environment. This implies facing and managing biological, psychological and social challenges and threats and remaining healthy by simultaneously adopting a health-related behavior in support of physiological regulation by allostatic and homeostatic adaptation. In other words, from a biopsychosocial perspective, maintenance of health requires autoregulatory capacity and complementing self-regulatory skills to sufficiently meet one’s own basic biological needs. At the same time, maintenance of health requires pursuing one’s own personal and social goals, expectations and ideals of well-being towards a fulfilled personal life within the limits set by one’s own social, ecological, economic and physical environment; however, while self-regulatory skills for contributing to one’s psychosocial well-being, such as social competence and emotional competence, are widely established, the same does not apply to self-regulatory body skills influencing one’s physical and mental well-being, health status and body functioning. Again, from a biopsychosocial model perspective, psychosomatic competence complements social and emotional competence as a third type of competence, which primarily addresses the physical aspect of the inseparable biopsychosocial unity [13].

**Fig. 1:**
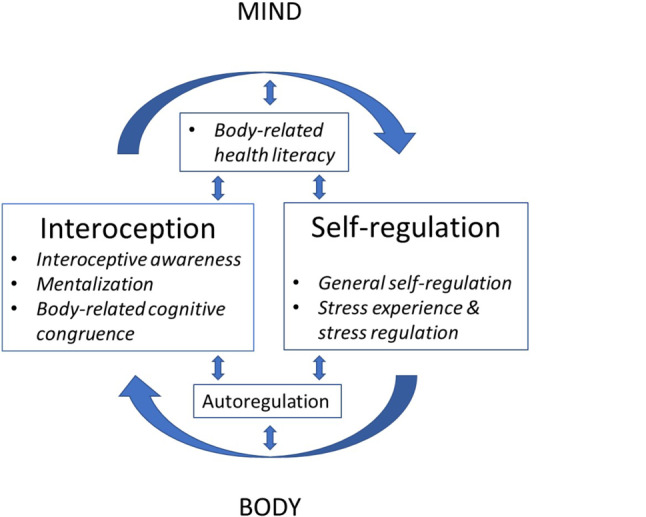
Psychosomatic competence model (PSC model). This model refers to a basic human ability to react consciously and adequately to perceived physical sensations by means of a central feedback-loop between interoception and self-regulation. All six components of psychosomatic competence (*in italics*) influence this feedback-loop: interoceptive awareness, mentalization, body-related cognitive congruence, body-related health literacy, stress experience and stress regulation, and general self-regulation. These six factors are intercorrelated and jointly contribute to psychosomatic competence as measured by the psychosomatic competence inventory (PSCI) [8]. In addition, the PSC model assumes a mutual influence between the interoception/self-regulation feedback-loop and autoregulation

The central element of the psychosomatic competence model (PSC model) is a feedback-loop between interoception and self-regulation monitored by cognitive factors. These factors are interoceptive awareness, mentalization of bodily states and an appraisal of physical states and signals termed body-related cognitive congruence. This cognitive appraisal results in interoceptive perceptions experienced as fitting or unfitting, understandable or inexplicable, pleasant or unpleasant, soothing or irritating, appeasing or demanding, disturbing or even unbearable. Interoception is known to contribute to the construction of the “self”. In accordance with recent neurobiological work on the self-in-context [5], both the concept and the model of PSC also assume that the construction of the self can influence body-related and health-related behavior towards the “self” in self-regulation [14]. In the PSC model self-regulation encompasses general self-regulation and stress-related self-regulation. This distinction was chosen since there is evidence that self-regulation behavior elicited by stressful experiences represents a specific self-regulatory area that differs, to a certain degree, from general self-regulation [8]. The PSC model is presented in Fig. 1. It shows the central feedback-loop between interoception and self-regulation as well as its six components (in italics) which influence the loop. These six components have been conceived based on theoretical considerations but their presence in the model is supported by empirical data [8].

## Terminology and significance of the components of the PSC model

### Interoception

In its current most widely used inclusive meaning, the term interoception refers to the subjective experience of the body state and is not restricted to any sensory channel [15]. Interoception indicates the set of perceptions of bodily signals and states, regardless of which type of information the brain uses or generates to construct this subjective experience related to the “material me” and the physiological condition of the body [16, 17].

According to the PSC model, the following three cognitive factors are linked to elaboration of essential clues provided by interoceptive signals and can thus be conducive to self-regulatory behavior, in particular in situations which are relevant for physical well-being and health-related behavior.

#### Interoceptive awareness

Up to now, there is no generally agreed taxonomy for *interoception science* and for the use of the term *interoceptive awareness* [18–20]. In a position paper on interoception and mental health it has been noted that the act of sensing, interpreting, and integrating information about the state of inner body systems can be related to different features, such as interoceptive attention, detection, discrimination, perceived intensity, accuracy, insight and sensibility [18]. The term interoceptive awareness has frequently been used to encompass any of the different interoception features accessible to conscious self-report. Regarding assessment of individual differences in interoceptive ability, Murphy et al. proposed a 2 × 2 factorial model in order to distinguish between what is measured (accuracy vs. attention), and how interoception is measured (objective measures vs. self-report) [20]. According to this, the meaning of interoceptive awareness in the PSC model corresponds largely to the concept of interoceptive attention, i.e. observing internal body sensations. Accordingly, the PSCI scale for interoceptive awareness primarily measures self-reported beliefs concerning one’s interoceptive attention, however, not as a general ability but in relation to specific situations in which interoceptive awareness could elicit self-regulatory behavior.

The PSC model assumes that interoceptive awareness raises attention to relevant information provided by interoceptive signals of the physiological state of the body. The PSCI as a self-report measure of psychosomatic competence uses several items related to interoceptive awareness, e.g. “I am consciously aware of sensations of physical pressure (e.g. an uncomfortable seat, a handshake)” or “I notice different physical responses depending on the setting (e.g., pleasant or unpleasant social surroundings).” Awareness of such body-related information may be conducive to self-regulatory behaviour, e.g., looking for and choosing a different seat while sitting on an uncomfortable one or taking a break from a fastidious activity or moving away from an unpleasant situation. In this context, it is relevant to note that self-regulatory behavior may also aim to reduce interoceptive awareness, e.g., by consciously distracting interoceptive attention from unpleasant physical symptoms, e.g., pain, itching, or hunger. Therefore, it can be summarized that in certain situations it may be helpful to become aware of interoceptive signals to trigger self-regulatory behavior and thus improve, e.g., physical well-being, while, in other situations, it may be preferable to be able to reduce interoceptive awareness and attention for the sake of physical well-being, e.g., during an unpleasant or painful medical procedure.

#### Mentalization

*Mentalization* describes the meta-cognitive ability to understand one’s own or other’s desires, feelings, attitudes, and beliefs based on behavior, i.e. to interpret invisible mental states by means of visible behavior. The mentalization concept is associated with a particular domain of the theory of mind research, a domain which examines the cross-culturally developing awareness in children of the fact that they themselves, or others, experience mental states “invisible” to the outer world [21]. Originally, the idea was introduced in the context of psychotherapeutic treatment and assumes that mentalizing ability develops through attachment experiences in childhood and can be qualitatively improved through psychotherapy [22, 23]. In contrast to this traditional understanding of mentalization with a focus on interpretation of behavior, the PSC model adds the ability of interpreting mental states also on the base of interoceptive signals. In other words, mentalization is the meta-cognitive ability to interpret mental states, which have their origin also in body-related information. Mentalization in this sense implies that physical changes can be accompanied by experiencing changes of mental states and vice versa. The PSC model assumes that interpreting interoceptive signals as connected with mental states can facilitate self-regulatory behavior. This assumption seems to be in line with functions attributed to self-in-context models as mentioned [5]. Mentalization in the PSC model proposes the ability to verbalize the perceived connection between interoceptive and mental states in a differentiated way. Examples of items directed towards measurement of mentalization in the PSCI are: “When I relax, I can sense physical changes” and “I can describe my various physical conditions (fitness, well-being, energy level) well using language.”

#### Body-related cognitive congruence

*Congruence/incongruence* are terms probably most frequently associated with Carl Rogers and their use in client-centered psychotherapy [24]. According to Carl Rogers, incongruence is a discrepancy between the perceived self and the actual experience of the organism [25]. In contrast, congruence describes a state in which a person’s perceived self and the actual experience are consistent or very similar. Rogers conceded that this state may be rarely fully achieved, and that people typically experience a certain amount of incongruence while, however, generally aspiring to congruence [26].

According to the PSC model interoceptive signals can either match or mismatch our expectations of these signals. In the former case they are experienced as congruent and contributing to the perceived self (“What I sense is making sense.”); however, a mismatch between interoceptive signals when compared to an expected or desired interoceptive state can be cognitively perceived as incongruent (something is going wrong, e.g. perception of getting sick in the beginning of a flu). This ongoing cognitive appraisal of interoceptive signals has been named *body-related cognitive congruence* in the PSC model. If interoceptive states or signals are perceived as incongruent, a search for causes and explanations for this incongruence may take place as well as possibly reactions aiming at restoring congruence. It is assumed that persons can use the perception of body-related congruence as health-related information. They may also use congruent/incongruent perceptions for self-monitoring of physical well-being and health-related behavior, in principle as a non-instrumental type of biofeedback signal provided by one’s own body. This ability may go as far as to anticipate and predict future interoceptive states related to specific situational circumstances as either congruent or incongruent [5, 7]. In addition, successful management of *body-related cognitive incongruence* may substantially contribute to maintaining health-related behavioral patterns, e.g., those related to eating habits.

Two examples for items of the PSCI scale body-related cognitive congruence are: “I am capable of recognizing the causes behind my physical sensations” and “When I exert myself physically, I can readily estimate how much I can demand of myself.”

### Self-regulation

In psychological research, various phenomena have been termed self-regulation and have been intermingled with the understanding of self-control. Nonetheless, there is a common agreement about the difference between the two concepts: Self-regulation has a broader meaning and refers to feedback loop models, or self-development and goal formation processes, whereas self-control has a narrower meaning and is more associated with impulse control and goal pursuit behavior [27–29]. There is clear evidence that lack of self-regulation in childhood leads to lifelong disadvantages in terms of unemployment, aggressive behavior, depression and anxiety, substance abuse, and symptoms of physical illness in adulthood [30].

Most publications to date have focused on mental and behavioral self-regulative processes, although first studies showed a clear connection between mental and body self-regulation [31, 32]. A framework presented earlier this year for analyzing the functional architecture of human motivation and personality functioning sees self-regulation as highly related to emotions and somatic information [33]. These self-regulatory processes are responsible for integrating implicit feelings, motives, and needs with consciousness and explicit identity formation. The conceptualization of psychosomatic competence (PSC model) is in line with such a comprehensive biopsychosocial approach to self-regulation but is primarily focused on the evidence of self-regulatory processes of somatic signals under normal conditions (general self-regulation) and stress conditions (stress experience and regulation). While developing the PSCI and in accordance with the best model fit of the questionnaire, the two conditions have emerged as related but separate conditions framing self-regulation [8].

#### General self-regulation

Examples of items for *general self-regulation* are: “I do not allow myself to be diverted from my original goals, even when I slip back into negative habits” and “At most I lose my composure only briefly, even when I cannot put an important plan into practice.”

#### Stress experience and stress regulation

Examples of items for *stress experience and stress regulation* are: “When I recognize that I have less energy than expected, I know why this is so” and “I can get myself going without stimulants (like coffee, for example) even when I am tired.”

### Feedback-loop between interoception and self-regulation

#### Body-related health literacy

The sixth component of the PSC model is related to the knowledge element and the self-management element of health literacy and was therefore named *body-related health literacy *[34]. It focuses on body-related personal knowledge that may influence the feedback-loop between interoception and self-regulation. Examples of items for body-related health literacy are: “I know exactly what I can do in order to feel physically well” and “I know which types of food I can tolerate well and which I cannot.”

#### Autoregulation

Although autoregulatory processes cannot be part of a human competence, autoregulation needs to be mentioned in this context. The PSC model assumes a mutual influence between the interoception/self-regulation feedback-loop and autoregulation, which includes homeostasis and allostasis. While homeostasis refers to a tendency towards a relatively stable equilibrium between interdependent elements, especially as maintained by physiological processes, e.g., regulation of body temperature, the concept of allostasis refers to the cumulative physiological changes that emerge by constant adaption to a variety of life experiences and stressors [35]. According to the concept of psychosomatic competence, there are many ways to influence unconscious autoregulatory processes. These range from very basic self-care, e.g., to regulate body temperature by dressing appropriately, to very specific body-related interventions, such as relaxation techniques for stress-reduction, biofeedback training, or physiotherapy; however, to the best of our knowledge there is to date no established general conceptualization and no established scientific term pointing to the human body-related ability to consciously trigger changes in autoregulatory processes by means of the feedback loop between interoception and self-regulation.

## Empirical evidence for the PSC model

### Related concepts

Empirical support for the concept of psychosomatic competence and its suggested favorable impact on health behavior change comes from disciplines that combine interoception and self-regulation as core elements of their practice, even if they may not explicitly refer to this theoretical construct and wording. One of these disciplines is mindfulness-based stress reduction (MBSR), others are often referred to as mind-body therapies and include for example Tai Chi, Qigong, Yoga, or meditation practices.

Mindfulness-based interventions are widely known and have been applied in several interventional studies [36, 37]. Empirical evidence suggests that they are associated with sustainable behavior change in a variety of health behaviors. This might be mediated via complex and synergistic effects of attention/cognitive control, emotion regulation and self-related processes, such as motivation and learning mechanisms. Details on these theoretical assumptions and health-related implications of MBSR have been recently reviewed [38].

Meditative physical practices (for instance Tai Chi and Qigong) have also shown positive effects on the performance of health behavior, such as diet behavior or stress management [39–41].

Perceived benefits of these interventions were manifold and included body-related effects, such as improvement of motor coordination, physical function or looseness, mind-related effects, such as improvements in self-efficacy and stress management and mind-body-related effects, such as increase of body awareness, relaxation or the ability to make self-corrections [42, 43].

These findings support the central element of the psychosomatic competence model which is the feedback loop between interoception and self-regulation, and point to the health-related potential for interventions addressing both body-related and mind-related pathways when initiating body-related and health-related behaviors.

Additional evidence is provided by the fact that through biofeedback training, self-control over physiological processes which are otherwise outside awareness or under less voluntary control can be achieved [44].

### Correlations of the PSCI scales

The development and first validation of the PSCI also provides preliminary evidence for the PSC model. As theoretically assumed, all six scales of the PSCI were shown to be significantly intercorrelated. These correlations based on factor scores resulting from item response theory (IRT) estimations ranged from 0.550 (lowest correlation between interoceptive awareness and stress experience and stress regulation;) to 0.706 (highest value between interoceptive awareness and mentalization) [8]. In a first validation of the PSCI all scales were positively correlated with self-efficacy and three PSCI scales (general self-regulation, stress experience and stress regulation, body-related health literacy) were negatively correlated with the number of reported bodily complaints as a measure of physical well-being.

## Conclusion and outlook

Research on psychosomatic competence requires an interdisciplinary approach. Both physiological and psychological avenues offer detailed insights which are, however, limited to one side of a two-sided matter; it seems clear that one side cannot be fully understood without the other. Empirical evidence of links between the two sides remains to date scanty. Many open research questions need to be clearly defined and investigated using modern perspectives and methods.

One particular open question is to what extent cognitive load and allostatic load impair psychosomatic competences. From psychological research, we know that prolonged stress and demanding situations lead to a weakening of self-regulatory abilities, starting with a loss of action control [45], phenomena called self-infiltration, in which people lose access to their own feelings and needs and can neither perceive nor communicate them [46, 47]. This may end with a loss of discrimination between wanted (own) and unwanted (foreign) goals [48]. It is reasonable to assume that stress also modulates body-related self-regulation, since the PSCI scales reach the best fit of the factor model when differing between general self-regulation and self-regulation under stress [8]. In clinical populations, burdensome and stressful interoceptive signals, such as chronic pain could similarly impair self-regulatory and health-related behavior. Future research aims at focusing not only on fostering psychosomatic competence with all its aforementioned trainable facets but also on processes, which can impair already acquired psychosomatic competence, as for example, cognitive or allostatic overload, chronic stress or chronic pain. Finally, as a primary research objective the assumed positive impact of psychosomatic competence on health-related behavior and health outcomes needs to be investigated and differences in psychosomatic competence and its components in specific clinical cohorts and in the healthy population need to be explored. To date, these are white spots on the research map.

However, more general research questions remain unacknowledged but potentially open up a new research realm, which directly focuses on the mind-body problem, one of the major philosophical and scientific problems. A first step to investigate this relationship with new methods, could be the more detailed exploration of phenomena of a regulatory nature, studied simultaneously at the physiological and psychological levels. One problem-solving approach is provided by the adaptation processes assimilation and accommodation, which are traditionally used to explain both biological and psychological phenomena [49]. Assimilation and accommodation help to understand how to cope with growing old [50], or to develop psychological resilience [51], but they are also applied to understand memory at the physiological level, in terms of the organization and function of place cells [52] or to investigate gene expression plasticity on the basis of genetic assimilation and accommodation, as it is discussed in evolutionary biology [53, 54]. Assimilation and accommodation, as fundamental processes of adaptation and regulation in living systems, hold great potential for a more sophisticated understanding of the mind-body connections or the mind-body unity [55].

The aim of the paper is bifold. First, the PSC model is presented with broadened theoretical embedding and recent empirical evidence. Second, it argues a desideratum of psychosomatic competence research. Research done over years identified the pivotal facets of psychosomatic competence, such as self-regulation under normal and stress situations, interoceptive awareness, mentalization, body-related cognitive congruence and body-related health literacy. So far, measurement possibilities could be developed and validated with concepts close to psychosomatic competence; however, many research questions remain open, some of them can be framed concretely, some of them still need high scientific effort on a theoretical and empirical level. Since psychosomatic phenomena of interest are complex and endemic in different disciplines, it is arguable to work on them in an interdisciplinary approach, on a psychological and psychiatric level but also on the level of other medical disciplines, such as cardiology, endocrinology, dermatology or physical medicine and rehabilitation.
